# Collagen Organization Does Not Influence T-Cell Distribution in Stroma of Human Pancreatic Cancer

**DOI:** 10.3390/cancers13153648

**Published:** 2021-07-21

**Authors:** Eva-Maria Kamionka, Baifeng Qian, Wolfgang Gross, Frank Bergmann, Thilo Hackert, Carlo A. Beretta, Nicolas Dross, Eduard Ryschich

**Affiliations:** 1 Department of General, Visceral, and Transplantation Surgery, Heidelberg University Hospital, Im Neuenheimer Feld 365/420, 69120 Heidelberg, Germany; e.kamionka@googlemail.com (E.-M.K.); mike1232006@123.com (B.Q.); wgross@uni-hd.de (W.G.); Thilo.Hackert@med.uni-heidelberg.de (T.H.); 2 Department of Pathology, Heidelberg University Hospital, Im Neuenheimer Feld 224, 69120 Heidelberg, Germany; frank.bergmann@med.uni-heidelberg.de; 3 CellNetworks Math-Clinic, University of Heidelberg, Bioquant BQ001, Im Neuenheimer Feld 267, 69120 Heidelberg, Germany; carlo.beretta@uni-heidelberg.de; 4 Institute for Anatomy and Cell Biology, University of Heidelberg, Im Neuenheimer Feld 307, 69120 Heidelberg, Germany; 5 Nikon Imaging Center, University of Heidelberg, 69120 Heidelberg, Germany; nicolas.dross@bioquant.uni-heidelberg.de

**Keywords:** human pancreatic cancer, collagen organization, T-cell infiltration, tumor stroma, chemokines

## Abstract

**Simple Summary:**

The excessive desmoplasia is the hallmark of human pancreatic cancer that influences the local T-cell-based immune response. In the present work, the stromal collagen organization in normal and malignant pancreatic tissues as well as its relationsship to T-cell distribution in pancreatic cancer were studied. It was found that differences in collagen organization do not change the spatial orientation of T-cell migration and do not influence the availability of tumor cells for T-cells. The results of the study do not support the concept of use of stroma collagen organization for improvement of spatial T-cell distribution in the tumor.

**Abstract:**

The dominant intrastromal T-cell infiltration in pancreatic cancer is mainly caused by the contact guidance through the excessive desmoplastic reaction and could represent one of the obstacles to an effective immune response in this tumor type. This study analyzed the collagen organization in normal and malignant pancreatic tissues as well as its influence on T-cell distribution in pancreatic cancer. Human pancreatic tissue was analyzed using immunofluorescence staining and multiphoton and SHG microscopy supported by multistep image processing. The influence of collagen alignment on activated T-cells was studied using 3D matrices and time-lapse microscopy. It was found that the stroma of malignant and normal pancreatic tissues was characterized by complex individual organization. T-cells were heterogeneously distributed in pancreatic cancer and there was no relationship between T-cell distribution and collagen organization. There was a difference in the angular orientation of collagen alignment in the peritumoral and tumor-cell-distant stroma regions in the pancreatic ductal adenocarcinoma tissue, but there was no correlation in the T-cell densities between these regions. The grade of collagen alignment did not influence the directionality of T-cell migration in the 3D collagen matrix. It can be concluded that differences in collagen organization do not change the spatial orientation of T-cell migration or influence stromal T-cell distribution in human pancreatic cancer. The results of the present study do not support the rationale of remodeling of stroma collagen organization for improvement of T-cell–tumor cell contact in pancreatic ductal adenocarcinoma.

## 1. Introduction

Pancreatic ductal adenocarcinoma (PDAC) is an aggressive malignancy with a very poor prognosis [1]. Standardized therapies such as surgery, chemo- and radiotherapy have failed to cure this cancer in a great majority of patients. The mean 5-year survival of patients is less than 10% [2]. There is, therefore, an urgent need to develop more effective therapeutic strategies for the treatment of pancreatic cancer. 

Strong desmoplasia is a hallmark of pancreatic cancer [3], which can expand to more than 70% of the tumor tissues [4]. It has been well established that the tumor stroma plays an important role in the biology of this malignant disease. While the stroma in PDAC has a tumor-promoting effect owing to the activation of tumor cell invasion and metastasis [3,5], in contrast, depletion of tumor stroma can accelerate tumor progression owing to the loss of differentiation, induction of hypoxia, epithelial-to-mesenchymal transition, and the promotion of cancer stem cells [6]. 

T-cell movement through the three-dimensional (3D) extracellular matrix (ECM) occurs via a process of amoeboid-like migration [7]. It is driven by chemokines and is characterized by adaptive morphology, vigorous shape change, crawling along collagen fibrils (contact guidance), and squeezing through pre-existing matrix gaps [8,9]. In contrast to migration across two-dimensional (2D) substrates, it is usually independent of the interaction with integrins and matrix metalloproteinases [9,10]. Although 3D ECM creates a scaffold that supports 3D T-cell migration, a dense ECM fiber network can form non-permissive regions that exclude T-cell entrance. Such regions were demonstrated in the stroma of some human tumor types and have been proposed to weaken the local immune response [11,12]. Further, PDAC induces a cellular immune response [13,14]. Although direct contact between T-cells and tumor cells is important for prognosis [15], tumor-infiltrating T-cells do not reach tumor cells in sufficient numbers and can mainly be found in the tumor stroma at a distance from tumor cells [16,17]. 

Our previous study investigated the mechanisms controlling intratumoral T-cell migration in PDAC in terms of chemokines and contact guidance and determined whether and how these mechanisms contribute to immune evasion of pancreatic cancer [18]. Additionally, PDAC overproduced several T-cell-active chemokines, but their levels did not correlate with intratumoral T-cell infiltration [18]. Collagen itself promoted the high migratory activity of T-cells, but completely abolished chemokine-guided movement [18]. 

Generally, fibrillar collagen is highly abundant in the stroma of pancreatic cancer [4,19,20]. The local structure of collagen organization is the result of complex physiological processes, which include the activity of collagen-producing and -remodeling cells [21,22] and physical factors such as interstitial flow, spatial movement, and expansion of proliferating cellular components [23,24]. Several features such as fiber diameter, length, straightness, alignment (parallelism degree of single fibers), and spatial orientation to cellular structures as well as collagen density define the mechanical properties of the collagen network and potentially influence the movement of stromal cells. 

It has previously been shown that the alignment of collagen fibers is an important factor that controls the contact guidance of tumor cells [25,26]. In the stroma of human tumors, several patterns of collagen alignment have been recognized and proposed to promote tumor cell migration and invasion [27,28,29]. Previous studies have mainly addressed the effects of collagen alignment on tumor cells, with little focus so far being paid to T-cells. In contrast to tumor cells, migrating lymphocytes use short-lived, poorly adhesive cell–substrate interactions and do not degrade or reorganize the extracellular matrix, but rather slip through and along existing tissue gaps and trails [30,31]. Consistent with this fact, it can be proposed that collagen organization may influence T-cell migration and potentially contribute to stromal T-cell distribution. In the context of the emerging role of cancer immunotherapy, an examination of this hypothesis could have a high translational relevance, as the stroma is the microenvironmental factor that participates in local T-cell immune responses in pancreatic cancer [32]. An examination of the above hypothesis would help us to better understand the T-cell immune response, depending on the individual spatial and cellular organization of the tumor stroma. Furthermore, several previous studies have suggested stroma remodeling or stroma destruction for potential tumor treatment strategies [3,33]. An examination of the above hypothesis would provide important information that could support the rationale for these strategies. 

In the present study, we defined individual stroma regions in PDAC patients based on the fibrillar collagen network. Subsequently, the newly established procedure of image processing and analysis enabled, for the first time, the generation of tailored data on collagen organization and T-cell distribution in two individually defined PDAC regions. 

## 2. Material and Methods

### 2.1. Patients and Tissue Samples 

Tissue samples of the normal pancreas, chronic pancreatitis, PDAC, and ACC were obtained from the tissue collection (PancoBank) of the European Pancreatic Center (EPZ), University of Heidelberg. Patients admitted to the study were undergoing surgery for pancreatic diseases in the Department of Surgery at the University of Heidelberg. The protocol was approved by the local ethics committee and informed consent was obtained according to the Helsinki declaration. Tissue samples were snap-frozen and stored in liquid nitrogen. Hematoxylin and eosin staining of 7 µm slides was performed using Meyer’s hemalaun (Merck, Darmstadt, Germany) and Eosin G (Carl Roth, Karlsruhe, Germany). The histological diagnosis and grading of single tumor samples were performed by a specialist in pancreatic pathology (F.B.). The tumor cellularity of PDAC samples was analyzed as previously described [4]. It was defined as high (>30%) or low (≤30%) and was studied microscopically by subjective assessment of tumor cell fraction on histological slides (evaluated by E.R.). The tissue sample information is summarized in Table S1. 

### 2.2. Immunofluorescence Staining

All histological and immunofluorescence stainings were performed using filtered particle-free solutions and under sterile conditions, which ensured that optical artefacts did not appear in the tissue slides. For immunohistochemistry, 7 or 100 µm (for 3D reconstruction) acetone-fixed cryosections were pre-blocked using 20% goat serum (Agilent, Santa Clara, CA, USA) for 30 min and stained using rabbit anti-human Cytokeratin 7 (CK7, 50 ng/mL, clone EPR17978, Alexa Fluor 488-conjugated; Abcam, Cambridge, UK) and mouse anti-human CD3 (10 µg/mL, clone HIT3A; Biolegend, San Diego, CA, USA) followed by Alexa Fluor 568-conjugated secondary anti-mouse IgG (dilution 1 ÷ 200, Abcam). All incubations of labeled antibodies were performed in the dark for 1 h at room temperature. The staining procedures of 7 and 100 µm tissue slides were identical. Respective isotypic antibodies (Biolegend) were used as controls for immunofluorescence staining. 

### 2.3. Multiphoton and Second Harmonic Generation (SHG) Microscopy 

A TriM Scope 2-photon microscope (LaVision BioTec, Bielefeld, Germany) mounted on a Nikon FN-1 upright stand (Nikon Instruments, Düsseldorf, Germany) and equipped with a water-dipping 16× NA 0.8 long working distance objective (Nikon Instruments, Düsseldorf, Germany) was used. The excitation was carried out with a Chameleon Ultra II femtosecond titan:sapphire laser (Coherent, Dieburg, Germany) (80 MHz, 730–1050 nm) at 800 nm for collagen and 940 nm for Alexa Fluor 488-labeled epithelial cells and 850 nm for Alexa Fluor 568-labeled T-cells. The forward SHG signal of the collagen fibers was detected with a 405/10 nm bandpass filter. The emission of the labeled tumor clusters was detected at 525/50 nm and the emission of the labeled T-cells at 605/50 nm. Individual images of 625 µm × 625 µm (1611 px × 1611 px; 0.39 µm/px) were acquired with a 20% overlap between images. The interval between Z-stack images was set on 1.0 μm. Three-dimensional reconstructions of stitched stacks were created using arivis Vision4D software (arivis AG, Munich, Germany).

### 2.4. Pre-Processing and Computerized Analysis of Multiphoton and SHG Images 

#### 2.4.1. Segmentation of SHG and Multiphoton Images, CT-FIRE, and CurveAlign

**Figure 1:**
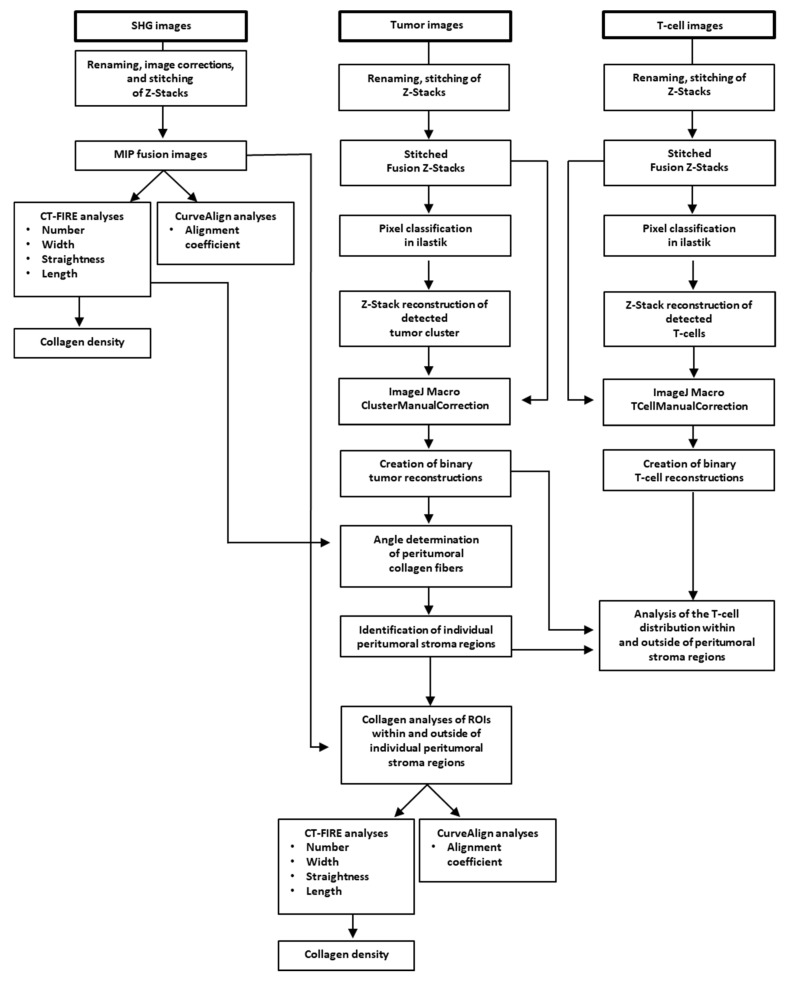
Image processing and analysis workflow of multiphoton and SHG microscopic data.

Image files of SHG, tumor, and T-cell images were renamed (macro RenameInputFile) in ImageJ plugin (NIH, Bethesda, MD, USA) and images were stitched (macro StitchingData) to create fused maximum intensity projection (MIP) images as well as fused Z-stacks. Prior to quantification of the collagen fibers, fused SHG MIP images were transformed into 8-bit images and adjusted using threshold between 10 and 255 gray levels to eliminate background noise. CT-FIRE and CurveAlign (LOCI, Madison, WI, USA) were run on fused MIP images according to the manufacturer’s instructions to calculate the collagen fiber parameters: length, number, straightness, and width (CT-FIRE V1.3); fiber alignment (CurveAlign V3.0), as previously described [34,35]. Fiber density was calculated by number of fibers per image area. The procedure is summarized in the workflow chart from Figure 1.

Tumor cluster and T-cell segmentation was performed using a combination of the pixel classification workflow based on the machine-learning image analysis (ilastik) [36] and ImageJ. The pixel classification workflow was used to enhance the foreground pixel and remove the background. Raw z-stacks of the tumor and T-cell images were used as input to train the pixel classification workflow. The ilastik machine-learning classifier was trained individually by creating for each single channel a pixel classification project. The training was performed on cropped (XY) images from different patients and applied by batch processing on the entire dataset of images. For those patients for whom the prediction did not match the training expectation, a new training session was performed by retraining the existing ilastik project with cropping (XY) of the problematic data or by creating a new ilastik pixel classification project trained on these images alone. The foreground probability map generated by ilastik for each input stack was processed in ImageJ. The binary masks of tumors and T-cells were extracted from the MIP and used further in the analysis (macros SegmentTumorCluster_ilastik.ijm, ManualClusterCorrection_ilastik.ijm, SegmentTCellAndManualCorrection_ilastik.ijm). For T-cells, a virtual particle filter was used, which eliminated all (non-cellular) particles below 25 pixels^2^. Indeed, the binary tumor MIP were used for the individual collagen analysis in the tumor microenvironment and the binary MIP of the T-cells for the individual T-cell distribution analysis inside and outside the peritumoral stroma regions of PDAC samples. The analysis steps are summarized in the ImageJ macros code (https://github.com/cberri/Kamionka_et_al-2020) and in the Table S2. 

#### 2.4.2. Definition of the Individual Peritumoral PDAC Stroma Regions

Images of tumor masks were generated by manually reconstructing the tumor clusters in fused SHG–MIP images using CurveAlign V4.0. The mean collagen fiber length in the PDAC samples was calculated to be 22.4 µm using CT-FIRE V1.3. Using these tumor mask images in combination with the previously generated CT-FIRE results of the average collagen fiber length in PDAC, a detailed, individual description of collagen in the PDAC microenvironment of twelve (see 4.4) selected PDAC tissues samples was generated. 

The mean fiber length (22.4 µm) was used as the interval by which the distance to microscopic tumor borderline was segmented. The analysis was performed in an individual area up to a maximal distance of 246.4 µm (eleven segments/areas of 22.4 µm). In these eleven areas, the angular distribution of the collagen fibers was analyzed and the relative fraction (R) of fibers having an angular range from 0° to 15° (aligned parallel to tumor cell borderline) was calculated. The ratio between individual R values and the mean (plateaued) R value from five distant areas ($\bar{R}$) was calculated using the formula:

(1) $$\frac{\left|R-\bar{R}\right|}{\bar{R}}$$

and was used for the definition of the extension of individual stroma regions. The areas showing a change in collagen orientation of at least 5% (ratio > 0.05) were assigned as tumor-distant stroma regions. If the ratio was <0.05, the area was assigned as a peritumoral stroma region. The determination of the above stroma regions is illustrated in Figure 6B.

#### 2.4.3. Detailed Analysis of Collagen and T-Cells in Different PDAC Stroma Regions

For gross analysis of T-cell infiltration, the local density of CD3+ T-cells was assessed semi-quantitatively by a primary investigator (E.M.K) using the scale: 0 = absent, 1 = low, 2 = moderate, 3 = high, and 4 = very high infiltration, as previously described [18] (Figure S1D). PDAC G1 tumors were not considered for further comparative analysis of T-cell infiltration because of low sample number. 

After identification of the expansion of individual peritumoral stroma regions, peritumoral and tumor-cell-distant ROIs of binary images of T-cell reconstructions were generated in ImageJ (macro DrawRectangularROIs). The number of generated ROIs varied between 2 and 12 for both peritumoral and tumor-cell-distant ROIs per SHG image. The size of the generated ROIs corresponded to the X and Y dimensions of the individual peritumoral stroma region. The collagen fibers of the individual ROIs were analyzed using CT-FIRE and CurveAlign and the mean values of the five collagen parameters were determined for each PDAC tissue. The T-cell area in each ROI was measured in ImageJ in an automated way. 

#### 2.4.4. T-Cell Distribution in PDAC Stroma

PDAC specimens showing distinct T-cell infiltration were used for the analysis. The total time required for analysis of single images was very different. Twelve images (12 different patients) requiring the lowest time for image processing were selected for the analysis. 

The areas of the individual peritumoral stroma regions of PDAC images and the areas of the tumor clusters in the peritumoral and tumor-cell-distant stroma regions were determined in ImageJ. In the binary images of T-cell reconstructions, the total area of CD3+ T-cells per image and the area of CD3+ T-cells inside and outside the individual peritumoral stroma regions were determined. The T-cell coefficients and T-cell densities of the two PDAC stroma regions were calculated using the four formulas below:

(2) $${T-\mathrm{cell}\;\mathrm{density}}_{\mathrm{peritumoral}}=\frac{{\mathrm{peritumoral}\;T-\mathrm{cell}\;\mathrm{area}\;(\mathrm{mm}}^{2})}{{\mathrm{peritumoral}\;\mathrm{area}\;(\mathrm{mm}}^{2})}\;\times \;100\;\%$$

(3) $${T-\mathrm{cell}\;\mathrm{density}}_{\mathrm{tumor}-\mathrm{cell}-\mathrm{distant}}=\frac{{\mathrm{tumor}-\mathrm{cell}-\mathrm{distant}\;T-\mathrm{cell}\;\mathrm{area}\;(\mathrm{mm}}^{2})}{{\mathrm{tumor}-\mathrm{cell}-\mathrm{distant}\;\mathrm{area}\;(\mathrm{mm}}^{2})}\;\times \;100\;\%$$

(4) $${T-\mathrm{cell}\;\mathrm{coefficient}}_{\mathrm{peritumoral}}=\frac{\left[\frac{{\mathrm{peritumoral}\;T-\mathrm{cell}\;\mathrm{area}\;(\mathrm{mm}}^{2})}{{\mathrm{total}\;T-\mathrm{cell}\;\mathrm{area}\;(\mathrm{mm}}^{2})}\right]}{{\mathrm{peritumoral}\;\mathrm{area}\;(\mathrm{mm}}^{2})}$$

(5) $${T-\mathrm{cell}\;\mathrm{coefficient}}_{\mathrm{tumor}-\mathrm{cell}-\mathrm{distant}}=\frac{\left[\frac{{\mathrm{tumor}-\mathrm{cell}-\mathrm{distant}\;T-\mathrm{cell}\;\mathrm{area}\;(\mathrm{mm}}^{2})}{{\mathrm{total}\;T-\mathrm{cell}\;\mathrm{area}\;(\mathrm{mm}}^{2})}\right]}{{\mathrm{tumor}-\mathrm{cell}-\mathrm{distant}\;\mathrm{area}\;(\mathrm{mm}}^{2})}$$

### 2.5. Cell Culture and Activation of PBMLs 

Isolated peripheral blood mononuclear leukocytes (PBMLs) were isolated from the human blood of healthy donors using density gradient centrifugation with Biocoll (Biochrom GmbB, Germany). The cells were routinely cultivated in RPMI 1640 (cc-pro, Oberdorla, Germany) supplemented with 10% fetal calf serum (FCS) (cc-pro), 2 mM L-glutamine, 20 U/mL penicillin, 0.1 mg/mL streptomycin (cc-pro), 1 mM sodium pyruvate (Biochrom AG, Berlin, Germany), and 0.05 mM β–mercaptoethanol (Life Technologies, Darmstadt, Germany) at 37 °C in a humidified atmosphere with 5% CO_2_. 

For activation, PBMLs were stimulated with 12.5 µg/mL Concanavalin A (Calbiochem, Darmstadt, Germany) and 50 U/mL interleukin-2 (IL-2) (PeproTech, Hamburg, Germany) for day 1, and further with IL-2 for days 2–6. The activated cells were collected and used on day 6. To obtain the CD3+ population, mainly consisting of T-cells, the cells were labeled with PE-conjugated anti-CD3 mAb (clone HIT3a, BD Biosciences, Heidelberg, Germany) and isotypic mAb (clone G155-178, BD) was used as a control. The percentage of CD3+ cells was determined using a flow cytometer (FACS Canto II, BD Biosciences, Heidelberg, Germany) and FlowJo V.10.1 software (LLC, Ashland, OR, USA). The fraction of CD3+ cells was 70–80% (Figure S1C). 

### 2.6. T-Cell Migration in Collagen Matrix with Various Alignments 

For Boyden chamber assays, phenol red-free RPMI medium (MP Biomedicals, Solon, USA) supplemented with 1 mM sodium pyruvate (Biochrom, Berlin, Germany), 0.03 mM bovine serum albumin (fraction V, Carl Roth), 2 mM L-glutamine, 20 U/mL penicillin, 0.1 mg/mL streptomycin (cc-pro), and 0.05 mM 2-mercaptoethanol (Life Technologies) was used. The cells were labeled for 45 min at 37 °C with 4 µM calcein AM (Santa-Cruz, Dallas, TX, USA) and seeded in the upper wells (1 × 10^5^/well) of HTS Transwell-96 plates (Corning, Kaiserslautern, Germany). Migration was stimulated using 0–10,000 ng/mL SDF-1α, MIP-1β, or IP-10 (all from PeproTech) and measured after 3 h as fluorescence intensity in the lower wells using a spectrofluorimeter (FLUOstar OPTIMA, BMG Labtech, Ortenberg, Germany). This experiment was repeated four times. 

For the 3D collagen matrix, T-cells were fluorescently labeled using mouse anti-human CD3 (clone HIT3A, Biolegend) followed by Alexa Fluor 568-conjugated secondary anti-mouse IgG (Abcam). Approximately 1 × 10^6^ CD3+ T-cells were dissolved in a 200 μL solution consisting of concentrated RPMI-1640 medium (Sigma-Aldrich, Steinheim, Germany), supplemented with bovine serum albumin fraction V (BSA, Carl Roth) and 4-(2-hydroxyethyl)-1-piperazineethanesulfonic acid (HEPES, Carl Roth), 0.34 M sodium hydroxide, FITC-conjugated bovine collagen type I (Sigma-Aldrich), rat collagen type I (collagen R, 0.2% solution, Serva, Heidelberg, Germany), and magnetic beads (∅1 µm, chemicell, Berlin, Germany or ThermoFisher Scientific, Walham, MA, USA) (Table S3). The final collagen concentration was 0.95 or 1.5 mg/mL. The microfluidic chamber (μ-Slide VI, Ibidi, Martinsried, Germany) was filled with 90 μL/channel of the collagen/T-cell suspension. Additionally, SDF-1α (100 ng/mL), MIP-1β (100 ng/mL), or IP-10 (500 ng/mL) was added to some channels. The alignment of the fibers in the collagen matrices was achieved by generating a magnetic field using a cylindrical magnet (Conrad Electronics, Hirschau, Germany or Magnetladen GmbH, Schlangen, Germany) for 5 min each. The local magnetic flux density (4 mm distance from the magnet) was measured using Teslameter (CYHT208, ChenYang Technologies, Finsing, Germany) and was 45.5 and 50.0 mT for preparation of 0.95 and 1.5 mg/mL collagen, respectively. The collagen with T-cell suspension was polymerized at 37 °C and 5% CO_2_ atmosphere. 

For the recordings of the collagen matrices as well as the time-lapse videos of the T-cells, the Nikon A1R confocal microscope (Nikon Instruments, Düsseldorf, Germany) with a 20× immersion-free objective (Nikon, Plan Apo λ NA 0.75) was used. For imaging, the microfluidic chamber was placed into the temperature-controlled (37 °C) chamber (Tokai Hit, Shizuoka, Japan). Fluorescent collagens and T-cells were detected using excitations of 488 and 525 nm, and emissions of 525 and 595 nm, respectively. Time-lapse recording (2 images/min) of four neighboring fields (total surface 1.4 mm^2^) was performed for 30 min. The experiments were repeated six times, from which the data of T-cell migration without SDF-1α (22 or 23 channels for 0.95 or 1.5 mg/mL collagen, respectively) and with SDF-1α (18 or 22 channels for 0.95 or 1.5 mg/mL collagen, respectively) were collected. 

Time-lapse sequences were analyzed using ImageJ. The customized plugin Manual Tracking was used to determine the migration trajectories of single T-cells. The T-cell velocity was analyzed using the Chemotaxis and Migration software tool (Ibidi). The axial orientation of T-cell migration was measured as an integral value from single steps of individual T-cell trajectories. CT-FIRE and CurveAlign were run on fused confocal images to describe the collagen parameter in the in vitro model. Dependent on the local alignment coefficient, the collagen matrix was assigned to one of three groups: non-aligned, partially aligned, or aligned collagen (alignment coefficient <0.40, 0.40–0.60, or >0.60, respectively).

### 2.7. Statistics 

Statistical analysis was performed using the SPSS software (IBM, New York, NY, USA). The alignment coefficient of collagen fibers in the pancreatic tissue was compared using the *t*-test and Bonferroni–Holm correction. Differences in individual collagen parameters (alignment coefficient, length, diameter, straightness coefficient, and density) between different groups were compared using the Mann–Whitney U test and Bonferroni–Holm correction. A value of *p* < 0.05 was considered significant. Pearson’s correlation coefficient (r) was used to measure the strength of linear association between T-cell density and parameters of collagen organization in tissue as well as between collagen alignment and axial orientation of T-cell migration in vitro. 

## 3. Results

### 3.1. Collagen Organization and T-Cell Infiltration in the Stroma of Normal Pancreas, Chronic Pancreatitis, and Pancreatic Cancer 

#### 3.1.1. Normal Pancreas

**Figure 2:**
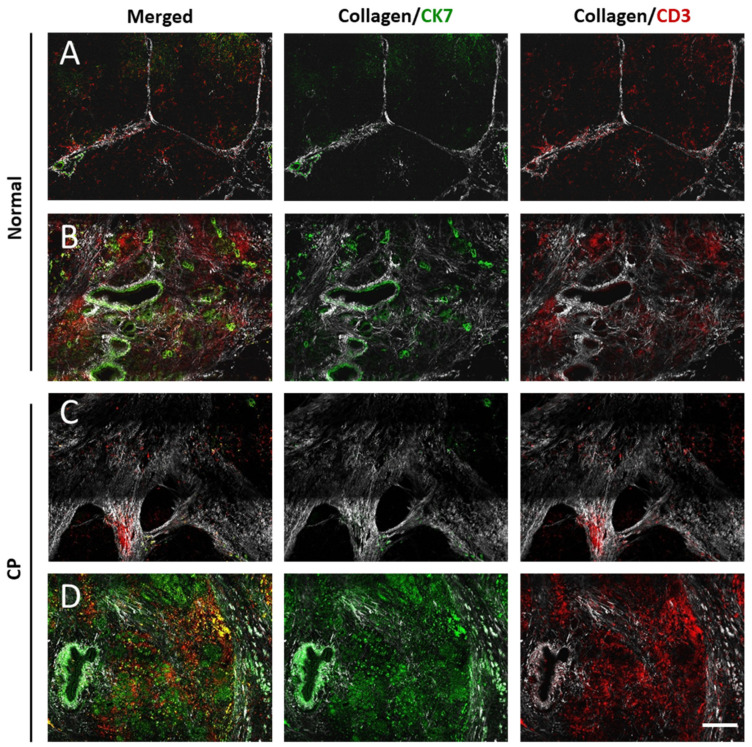
Representative microscopic images (fluorescence and SHG) of a normal pancreas and chronic pancreatitis. Collagen (white), CK7+ tumor cells (green), and CD3+ T-cells (red). Horizontal image lines show the same field of view with characteristic collagen pattern. *Normal pancreas*: (**A**) exocrine lobules, (**B**) interlobular stroma-rich region. *Chronic pancreatitis (CP):* (**C**) presenting high fibrotic tissue mass, (**D**) progressing exocrine degradation with strong inflammation. Scale bar 200 µm.

The SHG microscopy images showed perilobular collagen in the connective tissue (Figure 2A,B) and heterogeneous intralobular collagen distribution. A sparse periacinar collagen network, as well as collagen accumulation around periductal and perivascular structures, was observed (Figure 2A,B). The perilobular and intralobular collagen networks showed different forms. Perilobular collagen fibers of different lengths had a wavy or straight morphology and were largely in the same directional orientation. The intralobular collagen network was loosened, with wavy or straight collagen fibers of different lengths and inhomogeneous distribution. Concentric collagen organization was found in the periductal and perivascular areas as well as around pancreatic islands (Figure 2A,B). Some fibers in the perivascular area were also oriented perpendicular to the vessel wall. 

T-cells rarely infiltrated pancreatic tissues and formed local (mainly perivascular) accumulations of grades 1–2, and in a few cases of grades 3–4 (Figure 2B and Figure 4A). 

#### 3.1.2. Chronic Pancreatitis

Stroma in chronic pancreatitis was characterized by degradation of exocrine tissue and replacement of the parenchyma through the fibrotic tissue with high collagen content. Generally, collagen fibers in chronic pancreatitis followed the same organization as in normal pancreas (Figure 2C,D). Notably, regions of both dense and loosened collagen were found in the fibrous stroma (Figure 2C,D). 

Strong T-cell infiltration of grades 2–4 was found (Figure 4B). T-cells were mainly accumulated in perivascular areas and regions of loosened collagen (Figure 2C,D). 

#### 3.1.3. PDAC

**Figure 3:**
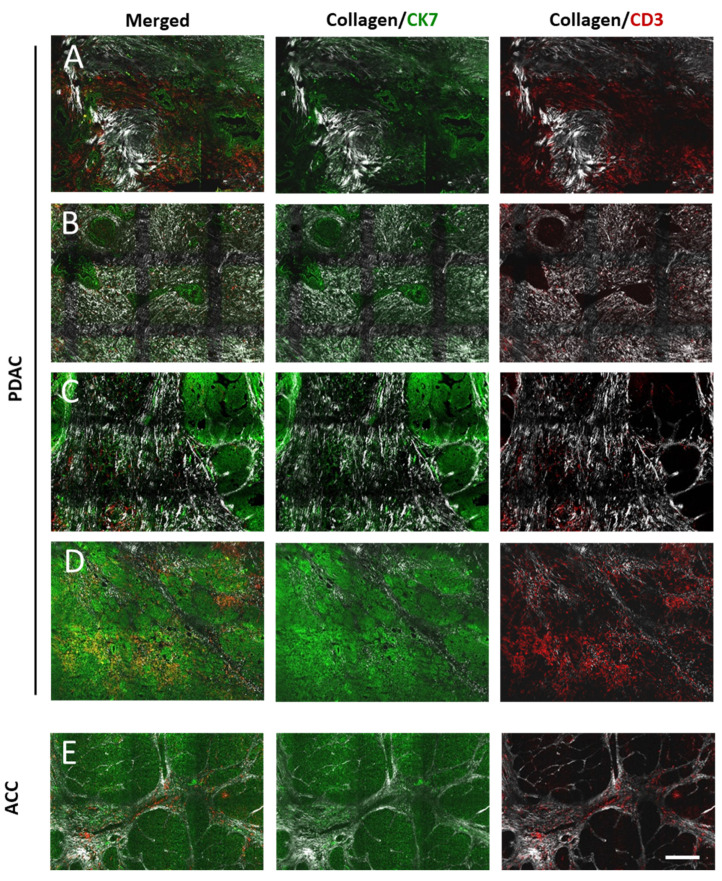
Representative microscopic images (fluorescence and SHG) of pancreatic cancer. Collagen (white), CK7+ tumor cells (green), and CD3+ T-cells (red). Horizontal image lines show the same field of view with characteristic collagen pattern. (**A**) *PDAC:* partially collagen-free regions and curved collagen fibers, (**B**) homogeneous well-aligned dense collagen, (**C**) well-aligned collagen with long fibers, (**D**) homogeneous loosened collagen, (**E**) *ACC:* collagen was detected in the narrow interlobular connective tissue and broad fibrous strands. Scale bar 200 µm.

Generally, the stroma of PDAC was characterized by a very heterogeneous collagen network with morphologically distinct collagen forms and organization patterns (Figure 3A–D). The collagen was inhomogeneously distributed within ECM. In PDAC, samples with free or partially collagen-free regions (Figure 3A) and regions with loosened (Figure 3B) or dense collagen (Figure 3C) were found. Several samples showed a network of homogeneous density both in peritumoral and tumor-cell-distant regions (Figure 3B,C). Other parts demonstrated inhomogeneous collagen distribution of different densities from missing/loosened to dense collagen (Figure 3A). In the peritumoral areas, collagen fibers were often wavy or straight. In these areas, long fibers were mainly oriented concentrically to tumor clusters, whereas short perpendicular orientation was found for short fibers (Figure 3A,B,D). In tumor-cell-distant areas, long, straight parallel-oriented and short, wavy, or straight chaotically oriented fibers were found. Furthermore, very long collagen fibers of several hundred micrometers crossing both peritumoral and tumor-cell-distant areas were detected (Figure 3B,C). 

**Figure 4:**
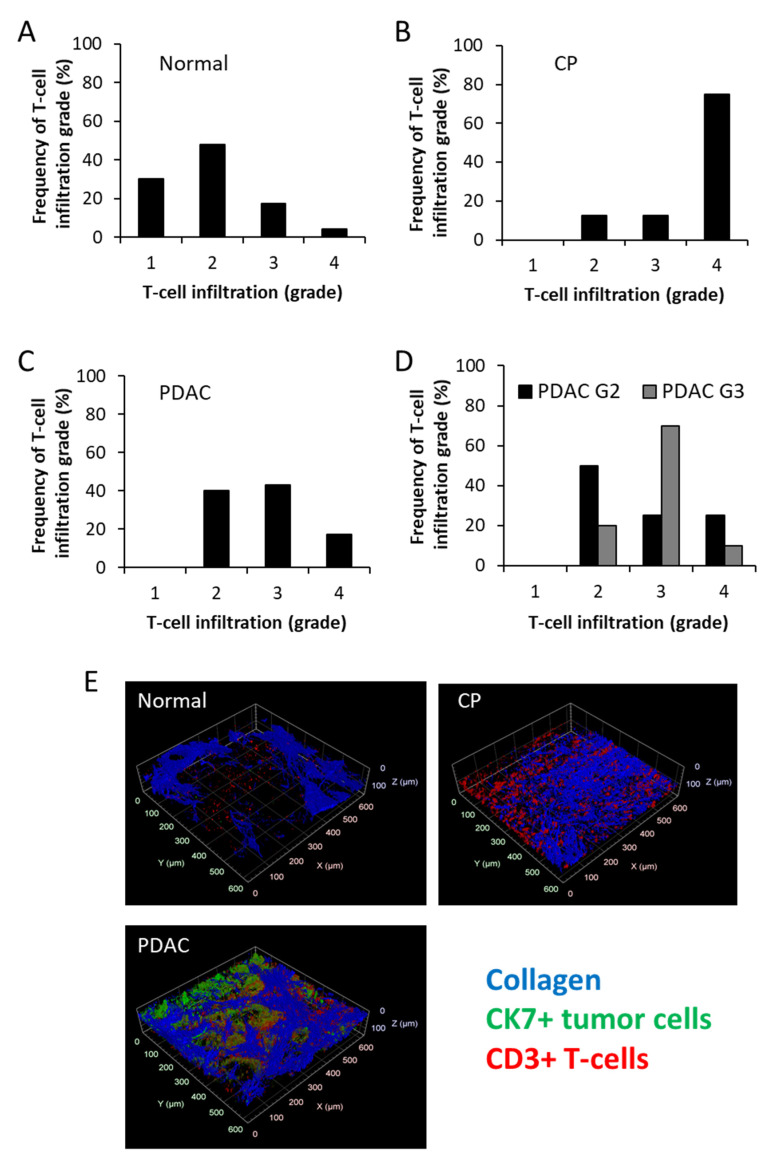
T-cell infiltration in different pancreatic tissue types. Grades of local T-cell infiltration in (**A**) normal pancreas, (**B**) chronic pancreatitis, CP, (**C**) total PDAC, and (**D**) according to G2/G3 PDAC. (**E**) Representative 3D reconstruction of fluorescence and SHG imaging.

Intratumoral infiltration of grades 2–4 were found in pancreatic cancers (Figure 4C,E). PDAC G3 showed more frequent infiltration of grades 3–4 when compared to PDAC G2 (Figure 4D). 

For the analysis of the spatial relationship between collagen, tumor cells, and T-cells, 3D reconstructions of multiphoton and SHG images were used. It was clearly demonstrated that T-cell infiltration was mainly localized in the stroma although the direct contact between T-cell and tumor cells could also be (in 23 of 32 samples) identified (Figure 4E). 

#### 3.1.4. Acinar Cell Carcinoma (ACC)

Two of three ACC samples showed minimal stroma having short collagen fibers with heterogeneous morphology (Figure 3E). In one sample, the better-developed stroma was dominated by broad fibrous strands, which contained long aligned collagen fibers. In the narrow interlobular connective tissues, collagen fibers showed two types of orientations: concentrically to tumor cell clusters or perpendicular, forming a rim around the tumor cell cluster border. 

ACCs were ubiquitously infiltrated with T-cells. Two samples showed moderate (grade 2), and one sample showed severe (grade 3) infiltration. Although direct T-cell–tumor cell contact, as well as tumor cluster-infiltrating T-cells, can frequently be found, the vast majority of infiltrating T-cells were localized in the stroma (Figure 3E). 

### 3.2. Quantitative Comparison of Collagen Organization between Different Tissue Types 

**Figure 5:**
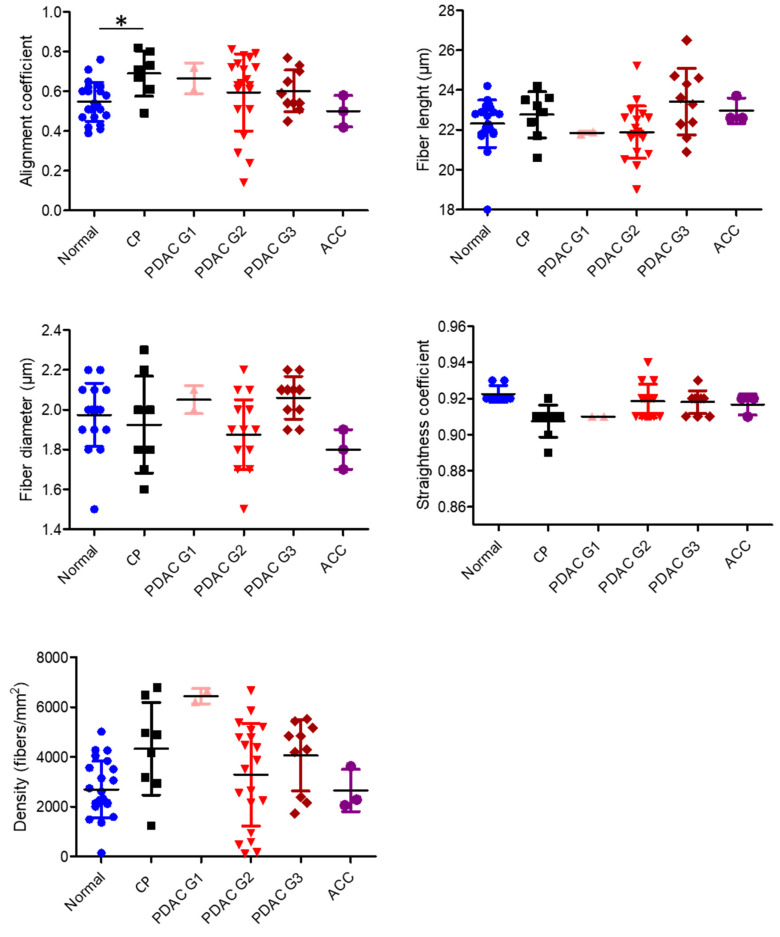
Quantitative analysis of stroma collagen organization. There were no significant differences in any parameters between any tissue types. Collagen alignment was significantly higher in chronic pancreatitis than in the normal pancreas. * *p* < 0.05, *t*-test.

Quantitative parameters of collagen organization were analyzed using SHG microscopy of histological slides and subsequent image processing. Mean collagen alignment and density showed heterogeneous values between individual specimens, whereas individual values of fiber length, diameter, and straightness were in comparable range (Figure 5). There were no significant differences in any parameters between any tissue types. However, only collagen alignment in chronic pancreatitis was significantly higher than that in normal pancreas, * *p* < 0.05 (Figure 5). There were no differences in any collagen parameters between G2 and G3 PDAC tumors (Figure 5). 

### 3.3. Collagen Organization and T-Cell Infiltration in Peritumoral and Tumor-Cell-Distant Stroma Regions in Pancreatic Cancer 

**Figure 6:**
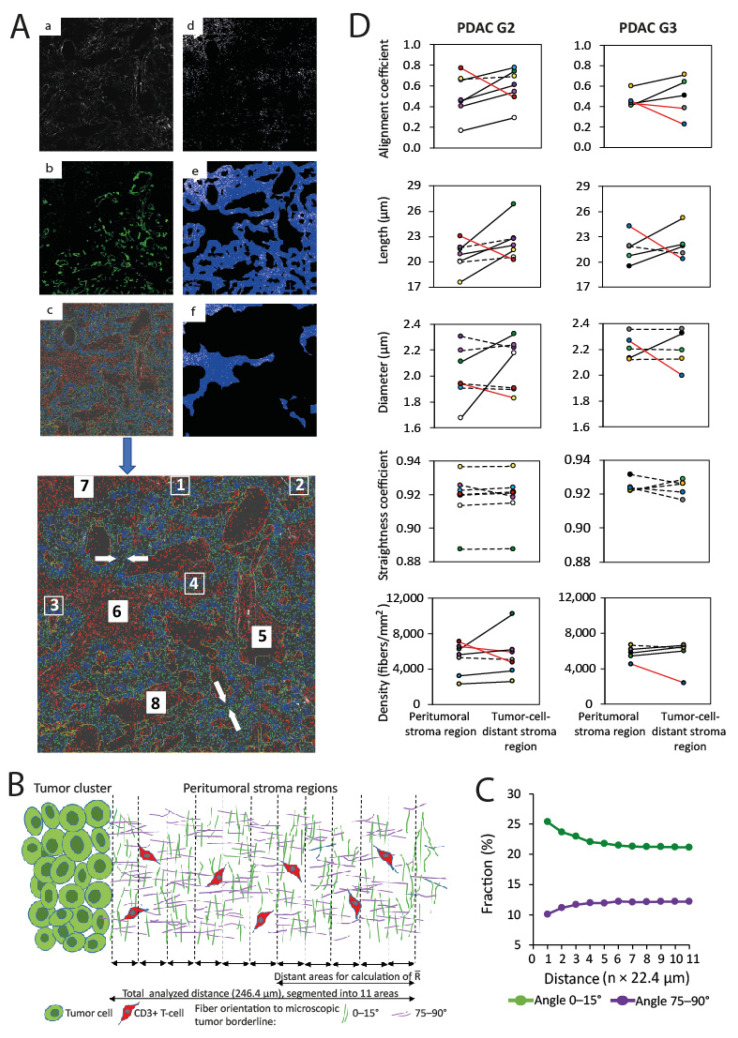
Definition and comparison of two individual stromal tumor regions in PDAC. (**A**) Illustration of segmentation steps and T-cell analysis. MIP-SHG fusion images of (**a**) segmentation of collagen surface and (**b**) CK7 + PDAC cells, (**c**) representative analysis of two stromal regions. Size-enhanced image of (**c**); the tumor cluster border is delineated by yellow line, the collagen fibers are marked in blue, and the collagen fiber ends are in green. The analyzed ROIs within the peritumoral stroma region are indicated by 1–4 and the ROIs of the tumor-cell-distant stroma region are indicated by 5–8. The arrows mark the border areas, which were excluded from the analysis because the distance between the tumor cell clusters is too short. (**d**) Determination of CD3 + T-cell area, which represents the base for the measurement of T-cell density in individual peritumoral (**e**) and tumor-cell-distant (**f**) stroma regions. T-cells are shown in white and the analyzed tumor-cell-distant stroma region is marked in blue. The image size is 1625 µm ×1625 µm. (**B**) Illustration of segmentation and definition of individual stroma regions. (**C**) Representative angular distribution of fiber orientation (Y-values show the fraction of parallel-oriented fibers) which is dependent on the distance to tumor cell clusters (X-axis, scaled in n multiples of 22.4 µm (mean fiber length)). (**D**) Paired values of parameters of collagen organization in PDAC between the peritumoral and tumor-cell-distant stroma regions. Black lines: increased values (change of >5%), red lines: decreased values (change of >5%), dotted lines: unchanged values (change of <5%).

Collagen organization in individual PDAC in relation to tumor cell regions was studied (Figure 6A,B). The study of collagen in all analyzed PDAC specimens demonstrated that its orientation was changed according to the distance from the borderline of tumor cell clusters: the shorter the distance to the tumor cell cluster, the higher the fraction of parallel-oriented fibers. This relationship was found in all analyzed samples, but only in regions that surrounded tumor cell clusters (Figure 6C). The extension of this region with changing collagen orientation was individual for each PDAC sample and ranged from 44.8–156.8 µm (Figure S1A). It was calculated using Formula 1 and was defined as the “peritumoral stroma region”. In contrast to “tumor-cell-distant stroma regions”, the peritumoral stroma regions in the majority of PDAC G2 were also characterized by decreased alignment coefficient and shorter fiber length (Figure 6D). Other collagen parameters such as fiber diameter, straightness, and density, did not show detectable differences between peritumoral and tumor-cell-distant stroma regions (Figure 6D). 

**Figure 7:**
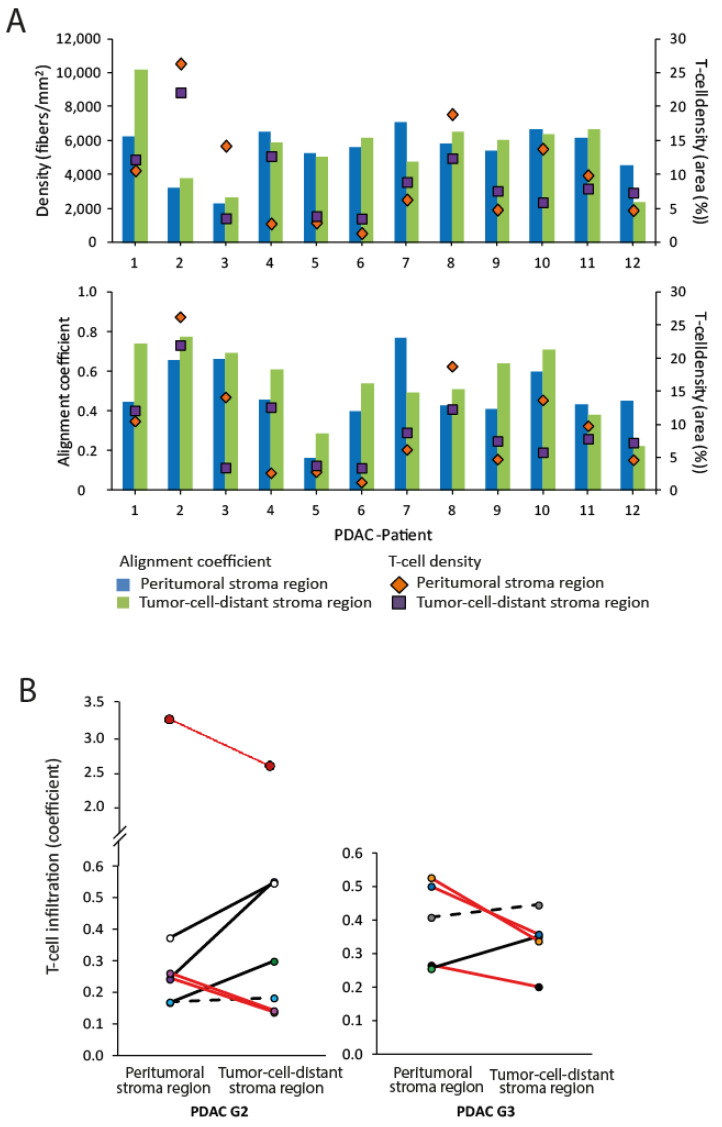
Comparison of collagen and T-cell distribution in PDAC. (**A**) No relationship between the collagen alignment/collagen density and the T-cell infiltration using the data of individual patients (*n* = 12) can be seen. (**B**) Paired values of the peritumoral and tumor-cell-distant stroma regions are shown. No significant difference was detected between the groups. Black lines: increased values (change of >5%), red lines: decreased values (change of >5%), dotted lines: unchanged values (change of <5%).

**Figure 8:**
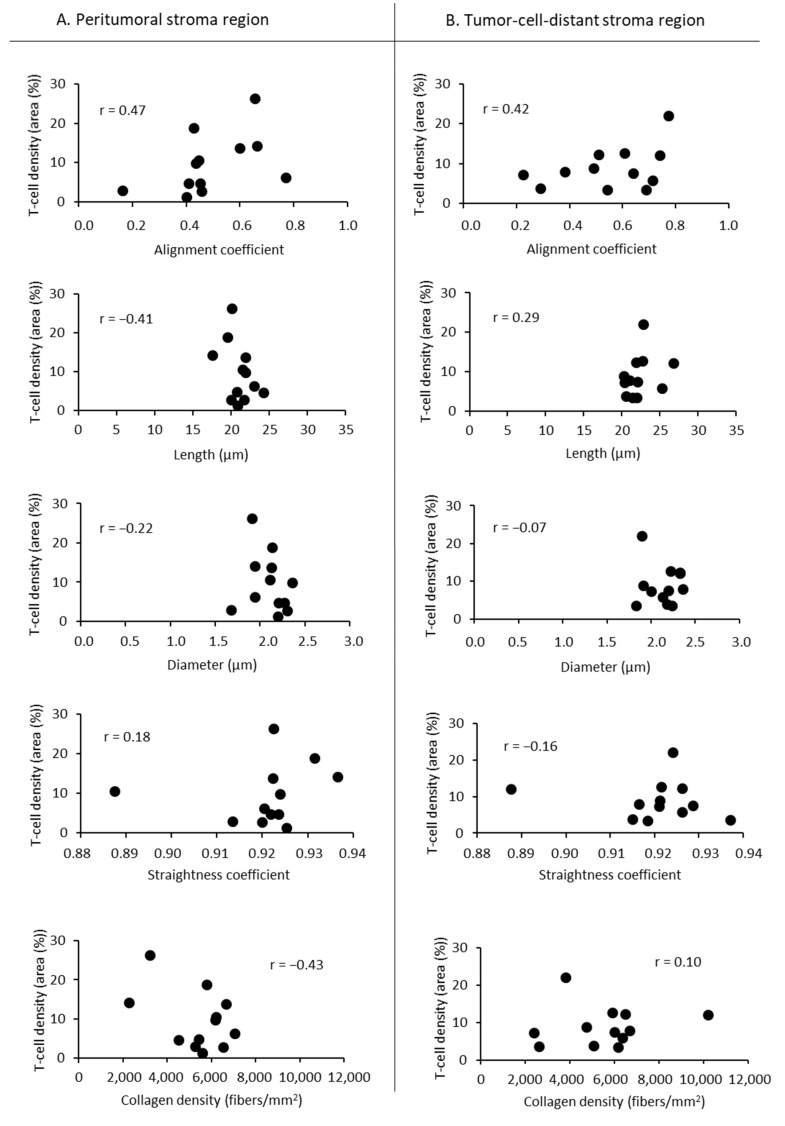
Correlation analysis between T-cell infiltration and single parameters of collagen organization in peritumoral and tumor-cell-distant-stroma regions. No correlations were found between T-cell density and collagen alignment, fiber length, fiber diameter, straightness coefficient, and collagen density. r, Pearson’s coefficient.

Although the collagen organization, in particular the collagen orientation, showed differences in the peritumoral and tumor-cell-distant stroma regions, there was no correlation in T-cell infiltration between these regions in all PDAC (Figure 7A) as well as in PDAC G2 and G3 tumors (Figure 7B). There were no correlations between T-cell density and any other parameters of collagen organization, such as alignment coefficient, fiber length, fiber diameter, straightness coefficient, and collagen density (Figure 8). 

### 3.4. Collagen Alignment Does Not Influence the Directionality of Activated T-Cell Migration in the Collagen Matrix 

**Figure 9:**
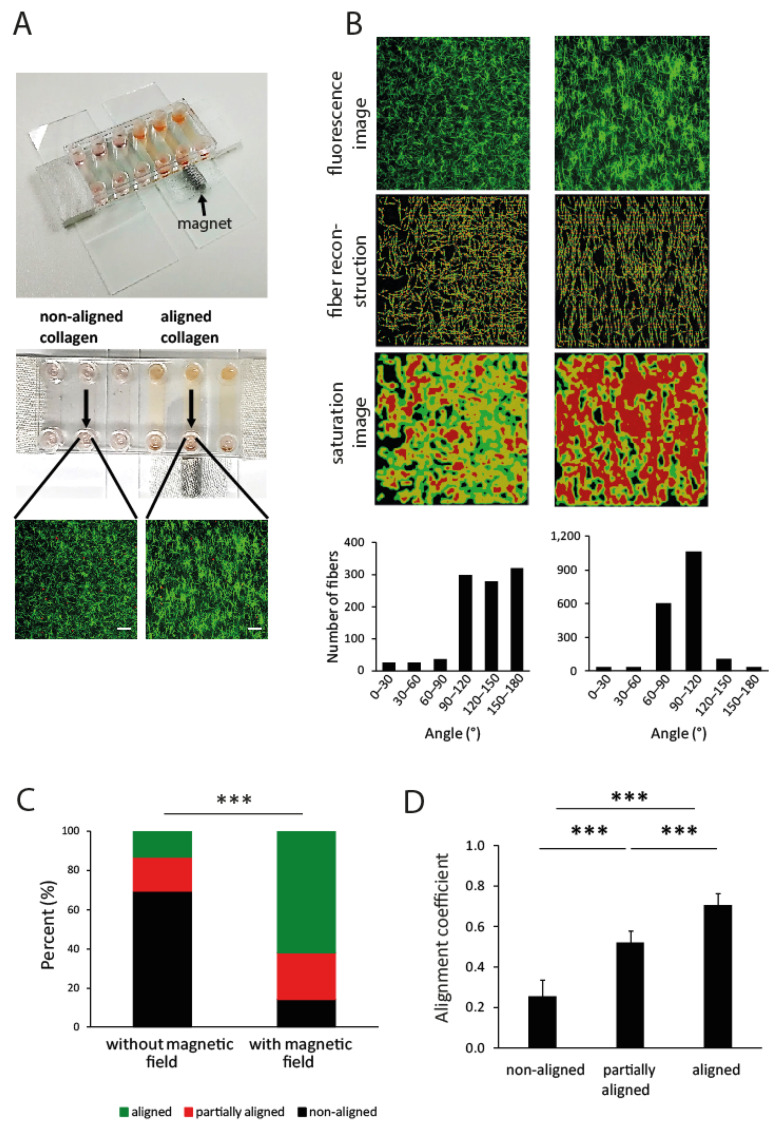
Design of 3D matrix with different grades of collagen alignment using the µ-Slide VI chamber. (**A**) Illustration of a single channel of the µ-Slide VI chamber for preparation of aligned collagen matrices using magnetic microparticles and a magnetic field. The arrows indicate the flow direction of the magnetic microparticles to the magnet. In confocal images, collagen fibers are shown in green and the T-cells are red. Scale bar 50 μm. (**B**) Representative determination of collagen alignment in one microscopic field. The illustration shows the collagen matrix (fluorescence) with or without alignment, the corresponding reconstructions of collagen fibers, saturation maps, and the angular distribution. The orientation angle of non-aligned collagen was broadly distributed, whereas the great majority of aligned collagen fibers showed the distribution at approximately 90°. (**C**) Distribution of collagen fiber alignment with and without magnetic microparticles. *** *p* < 0.001, Mann–Whitney U Test. (**D**) Mean alignment coefficient of collagen in the matrix. *** *p* < 0.001, Mann–Whitney U Test.

To verify the potential influence of collagen alignment on T-cell migration in vitro, the in vitro collagen model was utilized (Figure 9A). This model is based on collagen polymerization supported by the directional flow of magnetic beads produced by the oriented magnetic field (Figure 9A). Without a magnetic field, the partially directed collagen orientation and alignment occurs during the filling of the chamber, whereas the flow of magnetic beads further improves the collagen orientation and alignment (0.95 mg/mL collagen, Figure 9B–D, Figure S1B). 

**Figure 10:**
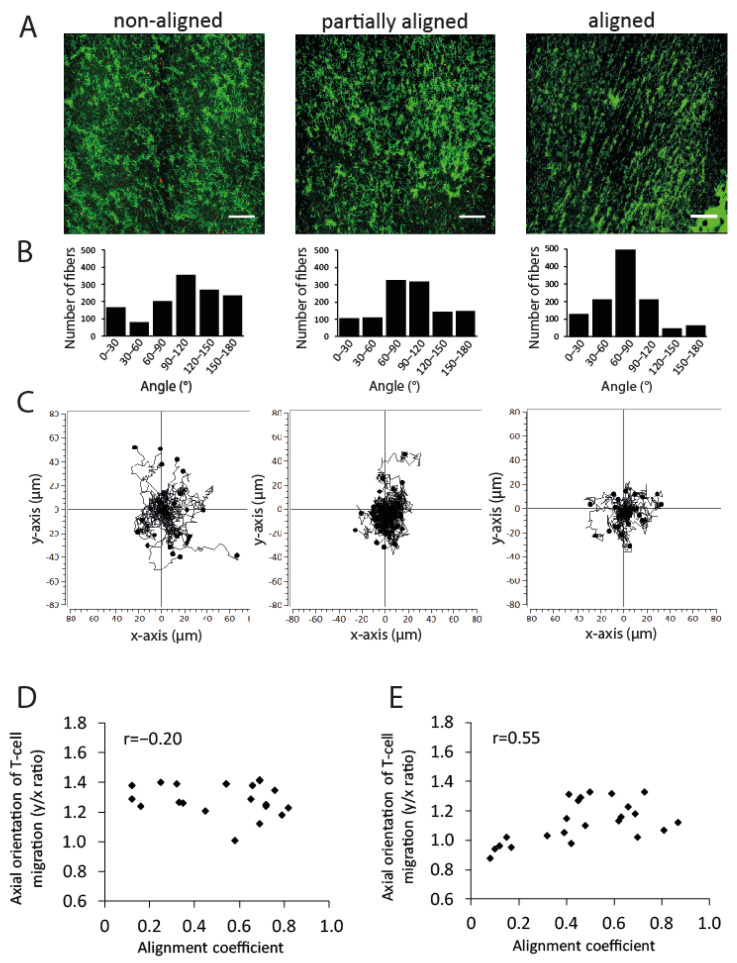
T-cell migration in the 3D collagen matrix. (**A**) Representative determination of T-cell migration in non-aligned, partially aligned, and aligned collagen matrices; 0.95 mg/mL collagen. Fluorescence collagen fibers (green), activated T-cells (red). (**B**) Angular distribution of collagen fibers (0.95 mg/mL collagen matrix) and (**C**) axial map of T-cell migration. (**D**,**E**) Axial orientation of T-cell migration in the matrix using 0.95 (**D**) or 1.5 mg/mL (**E**) collagen without chemokines. No relationship was detected between the alignment coefficient and axial orientation. r, Pearson’s coefficient.

**Figure 11:**
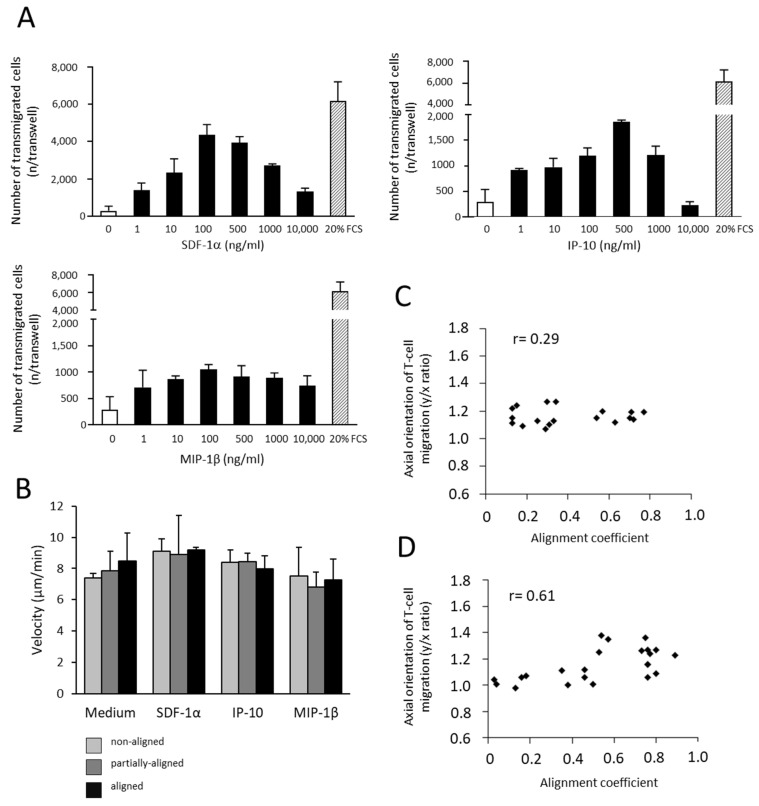
T-cell transmigration and haptokinesis in the presence of chemokines. (**A**) T-cell transmigration in a transwell assay using different concentrations of chemokines. T-cell transmigration showed high concentration-dependent sensitivity to chemokine stimulation. (**B**) T-cell haptokinesis in 3D-aligned collagen matrix model in the presence of chemokines. No effect of any chemokine and collagen alignment was found on T-cell velocity; 0.95 mg/mL collagen. (**C**,**D**) Axial orientation of T-cell migration in the matrix using 0.95 (**C**) or 1.5 mg/mL (**D**) collagen and SDF-1α (100 ng/ml). There was no relationship between the alignment coefficient and axial orientation. r, Pearson’s coefficient.

PBMLs were activated with Concanavalin A and IL-2 using the standard procedure, which produced cell suspensions with dominant (73.0 ± 5.7%) CD3 expression (Figure S1C). The T-cell migration was studied in collagen matrices of different alignment degrees (Figure 10A,B). It was found that the majority of the activated T-cells migrated actively and frequently changed direction (Figure 10C). Because the density represents the important mechanic property of the collagen network, T-cell migration in y/x orientation was analyzed using collagen matrices of two different densities. We found that there was no relationship between axial orientation of T-cell migration and collagen alignment using both 0.95 (Figure 10D) and 1.5 mg/mL (Figure 10E) collagen matrices. Chemokines showed a high chemotactic effect on T-cells in a transwell assay with variable concentrations of maximum stimulation (Figure 11A); however, no additive effect between the T-cell haptokinesis (velocity) and collagen alignment was found using the 3D collagen matrix model (Figure 11B, 0.95 mg/mL collagen). The axial orientation of T-cell migration in the aligned collagen matrix in the presence of SDF-1α was also not changed using both 0.95 (Figure 11C) and 1.5 mg/mL (Figure 11D) collagen matrices. 

## 4. Discussion

In the present study, the collagen organization in normal pancreas, chronic pancreatitis, and pancreatic cancer and its potential influence on the distribution of infiltrating T-cells was investigated. Two features of collagen organization, mean fiber diameter and straightness, were at the same range between different tissue types and also showed low individual deviations. The mean fiber length was also at the same range, although single fibers were very long. Because there were no differences, the above-mentioned parameters can hardly be expected to change T-cell migration. Other features, such as collagen density, alignment, and spatial orientation, showed higher individual heterogeneity and are discussed below. 

In a normal pancreas tissue, the stroma is formed during body growth and no significant remodeling is expected under healthy conditions. Therefore, stromal collagen in a normal pancreas displays a well-aligned and generally homogeneous structure of any determined parameters. Chronic pancreatitis showed the same homogeneous collagen organization as a normal pancreas. The microenvironmental changes in chronic pancreatitis develop slowly and, therefore, the collagen network in progressing fibrosis follows the same organization patterns as in the normal pancreas. 

In contrast to the normal pancreas, pancreatic cancer (PDAC and ACC) showed very heterogeneous patterns of collagen organization, although some common principles have also been found. Both PDAC and ACC may be considered as dynamic systems with continuously changing microenvironments, including spatial extension and active ECM production and remodeling. As expected, it had consequences for the collagen organization and resulted in an inhomogeneous collagen presentation, especially for fiber length, collagen density, and alignment. All these parameters could be important as a mechanical barrier for leukocytes; however, no significant differences in mean values between the normal pancreas, chronic pancreatitis, and pancreatic cancer were found. The only significantly increased collagen alignment observed was in chronic pancreatitis and may be the result of the high collagen productivity of cells in the fibrotic tissues. 

A group of PDAC samples showed a ubiquitous collagen organization, whereas other samples showed highly individual patterns. It could only reflect the inhomogeneity of local microenvironmental conditions (see above) influencing collagen production. 

Previous studies have analyzed the prognostic relevance of collagen organization and related it to changes in tumor invasion; in PDAC, high collagen alignment has been shown to be a negative prognostic factor [28]. Increased collagen fiber width is a negative prognostic factor in gastric cancer [29]. It has also been shown that the orientation of collagen bundles perpendicular to the tumor boundary (so called “TACS-3 signature”) facilitates local tumor invasion and represents a prognostic factor in human breast cancer [37,38]. This signature is of functional relevance to tumor cell invasion in breast cancer, but is not universal for other cancer types. Furthermore, in contrast to renal cell carcinoma [39], stromal collagen organization was not different in tumors of different grades of differentiation. 

Previous studies have established that pancreatic cancer induces T-cell immune responses; however, infiltrating T-cells are mainly “trapped” in the tumor stroma [16]. This definition was obtained using a steady-state view on histological slides. In the present study, we also found that a majority of the infiltrating T-cells were identified in the stroma and did not establish direct contact with the tumor cells. However, as it has been shown in a model of pancreatic cancer, intrastromal T-cells are actually actively migrating [40]. Therefore, the term “trapping”, that might mean “immobilization”, is not appropriate because it does not reflect the actual dynamic of T-cell behavior.

Among the different parameters of collagen organization, collagen alignment and its directional orientation were proposed to be important factors that could guide T-cell migration and distribution. In the present study, only the specific angular ranges of collagen fibers were dependent on the distance to the tumor stroma border by which peritumoral and tumor-cell-distant stroma regions were determined. We propose that this difference can mainly be caused by stroma compression through the spatial expansion of tumor cells. Interestingly, no relationship was found between the T-cell densities in the peritumoral and tumor-cell-distant stroma regions. It demonstrates that the spatial orientation of collagen did not change T-cell distribution in the stroma of pancreatic cancer. 

T-cell migration was also analyzed in differently aligned and differently dense collagens in an in vitro model. Although the phenotype of in vitro activated T-cells is different to that of tumor-infiltrating lymphocytes, this model could be representative of studies of the mechanical interaction between aligned collagen fibers and migrating T-cells. The rigid nucleus defines the smallest size of gaps or pores that can be breached by T-cells during migration [30,41]. The final structure and porosity of the matrix are dependent on several factors, including the origin of the collagen, its concentration, and the temperature of the polymerization process [30]. As shown in our previous study, 1.5 mg/mL collagen strongly inhibited the entrance of T-cells into the matrix, whereas collagen at a lower concentration (1 mg/mL) supported the effective invasion of migrating T-cells [18]. In the present study, no relationship was found between the angular orientation of T-cell migration and collagen alignment both in soft and dense collagen matrices. Although chemokines (SDF-1α, IP-10, and MIP-1β) strongly attracted T-cells in a collagen-free system, they showed no effect on the velocity and orientation of T-cell migration in the collagen matrix. These results are consistent with data from a previous study, which demonstrated that collagen alignment guides the direction of mesenchymal, but not of amoeboid-like cell migration [42]. 

Recently, Pruitt et al. reported a study on the guidance of activated T-cells by aligned collagen [43]. In this study, the authors prepared the matrix from collagen solution which was allowed to nucleate (to polymerize) for 2 h on ice and injected into the narrow microfluidic channel [43]. Because polymerized collagen matrix is very fragile [44], any further handling such as injection damages the integrity of the collagen network and may produce collagen gel with a ruptured structure. This could also be the reason why T-cell migration was observed even in very dense collagen gels (3 mg/mL), which are usually unbreachable by T-cells [30]. We believe that a more appropriate model was used in the present study because collagen polymerization occurred simultaneously with collagen alignment. 

Finally, the present study described the unique workflow, which included a complex of histological, microscopic, and image processing operations. The above operation algorithm enabled a more accurate analysis of pancreatic stroma collagen organization compared to the ones reported in any previous studies [45,46]. The workflow includes the processing of SHG images and analysis of collagen parameters using the ImageJ, CT-FIRE, and CurveAlign tools. This procedure has been previously described and utilized in numerous studies for the quantification of collagen organization [28,29,39]. On the other hand, machine-learning-based bioimage analysis (ilastik) [36] was used for pixel classification and segmentation of tumor clusters and T-cell areas and was additionally supported by several self-generated macro tools. This part of the workflow was created specifically for the aims of the present study. As shown in the present study, the procedure was applicable at different micro-regional levels, such as analysis of the whole slide surface, identification and analysis of the total surface in peritumoral and tumor-cell-distant stroma regions, and detailed analysis of selected regions of interest (ROIs). Furthermore, it excluded the majority of optical- and processing-related artefacts and enabled a very exact quantitation of T-cell infiltration using the determination of local T-cell areas, the calculation of T-cell density, and T-cell coefficient. The computer-aided determination of T-cell areas is superior for the exact calculation of T-cell density and can be used even for very dense T-cell infiltrates in which the precise counting of single T-cells would be almost unachievable. The present workflow provides a much more accurate analysis of collagen density in relation to tumor and T-cell area than the gross method reported in our previous study [18]. This study did not confirm the significance of collagen density for specific T-cell distributions in the stroma of pancreatic cancer. 

The low number of tissue samples could be a limitation of the present study; however, the total PDAC cohort (n = 32) should be sufficient for the characterization of collagen organization in this tumor type. Despite the use of machine-learning-based bioimage analysis, the workflow part of pixel classification and area analysis was a time- and resource-consuming procedure that in the present study could only be performed on a limited sample number. Nevertheless, the final number (n = 12) provided the information with the highest accuracy, and the data were conclusive for the described analyses. 

To summarize, in contrast with migrating tumor cells, collagen alignment does not force T-cells into directional migration, nor does it influence intratumoral T-cell distribution. From this point of view, the results of the present study do not support the rationale of remodeling of stroma collagen organization for improvement of direct T-cell–tumor cell contact in PDAC. The proposed tissue and image processing and analysis workflow may represent an excellent tool for the measurement of individual collagen organization in cancer patients. 

## Data Availability

Original data can be made available upon reasonable request.

## Supplementary Materials

The following are available online at https://www.mdpi.com/article/10.3390/cancers13153648/s1, Figure S1: (A) Individual extension of peritumoral stroma regions in G2 and G3 PDAC. (B) Parameters of collagen organization in 3D matrices. * *p* < 0.05, ** *p* < 0.01 *** *p* < 0.001, Mann-Whitney-U Test. (C) FACS analysis of CD3 expression after activation of PBMLs. Isotype (gray), anti-CD3 mAb binding (red). (D) Representative images of grades 2–4 of T-cell infiltration. Images represent: grade 2 (normal pancreas), grade 3 (pancreatic cancer), grade 4 (chronic pancreatitis). Collagen (white), CK7+ epithelial cells (green), CD3+ T-cells (red). Scale bar 100 µm, Table S1: Summary information of tissue samples, Table S2: Summary of macros tailored for computerized analysis of multiphoton and SHG images, Table S3: Composition of collagen matrices.
