# Supplementary material for: Collagen Organization Does Not Influence T-Cell Distribution in Stroma of Human Pancreatic Cancer
Source: Cancers (Basel). 2021 Jul 21;13(15):3648. doi: 10.3390/cancers13153648 (PMC8344977; doi:10.3390/cancers13153648)
Supplement: Supplementary file 1 [file cancers-13-03648-s001.zip › cancers-1248543-supplementary.pdf]

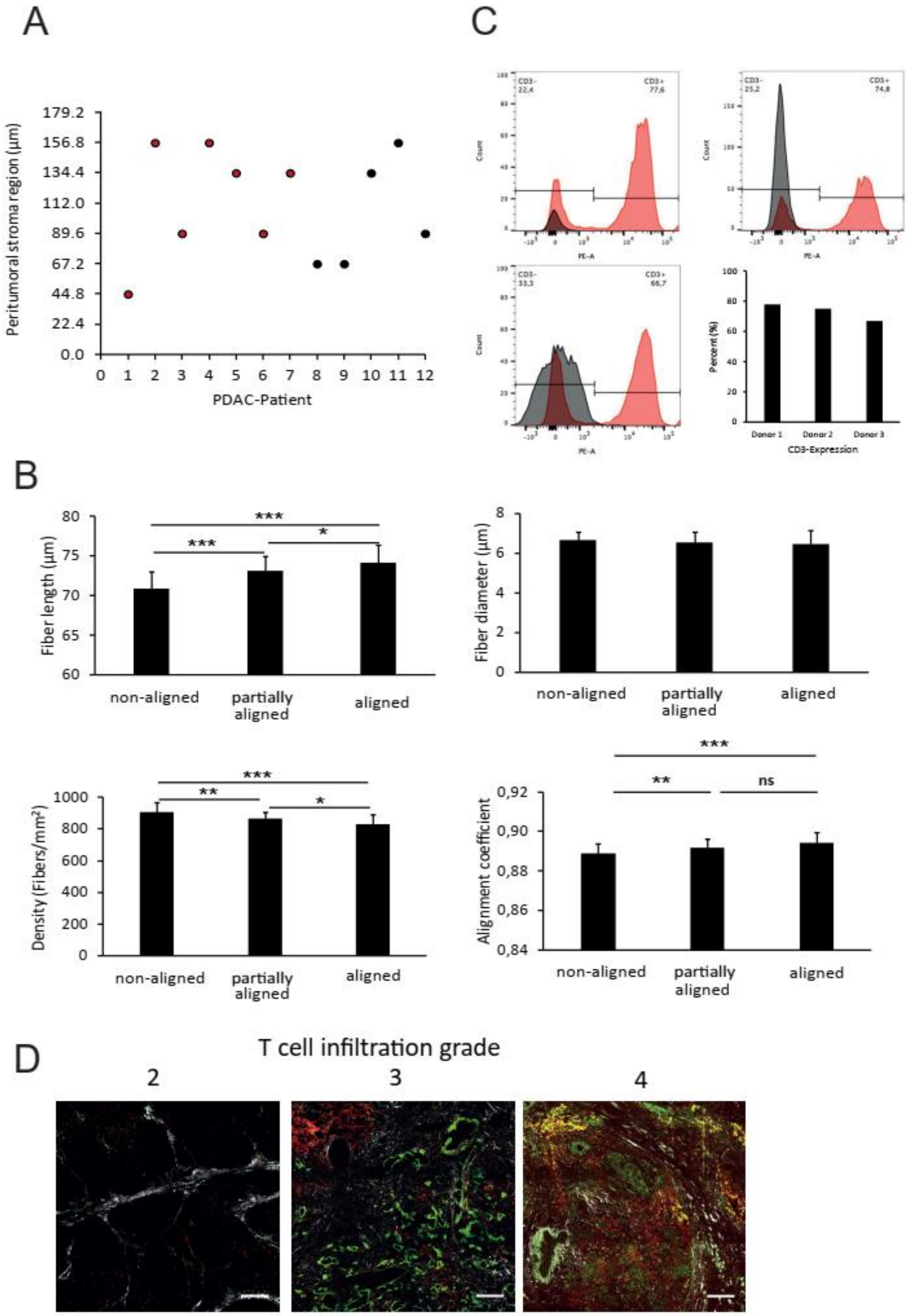

Figure S1 (A) Individual extension of peritumoral stroma regions in G2 and G3 PDAC. (B) Parameters of collagen organization in 3D matrices. * $p < 0.05$, ** $p < 0.01$ *** $p < 0.001$, Mann-Whitney-U Test. (C) FACS analysis of CD3 expression after activation of PBMLs. Isotype (gray), anti-CD3 mAb binding (red). (D) Representative images of grades 2–4 of T-

cell infiltration. Images represent: grade 2 (normal pancreas), grade 3 (pancreatic cancer), grade 4 (chronic pancreatitis). Collagen (white), CK7+ epithelial cells (green), CD3+ T-cells (red). Scale bar 100 µm.

Table S1. Summary information of tissue samples:

Normal pancreas:	
Age (years)	59 ± 12
Gender:	
Female	13
Male	10
Total	23
Chronic pancreatitis:	
Age (years)	59 ± 15
Gender:	
Female	6
Male	2
Total	8
ACC:	
Age (years)	55 ± 11
Gender:	
Female	1
Male	2
Total	3
PDAC:	
Age (years)	66 ± 8
Gender:	
Female	15
Male	17
Total	32
Grading:	
G1	2 (6.3%)
G2	20 (62.5%)
G3	10 (31.3%)
Tumor cellularity:	
High (>30%)	10 (31.3%)
Low (≤30%)	22 (68.8%)

Table S2. Summary of Macros tailored for computerized analysis of multiphoton and SHG images

ImageJ macros	Function	Author
RenameInputFile	Generation of uniform file names and folder structure	Carlo A. Beretta
StitchingData	Image tails stitching	Carlo A. Beretta
SegmentTumorClusterIlastik	Identification of tumor tissue	Carlo A. Beretta
ManualClusterCorrectionIlastik	Generation of tumor masks	Carlo A. Beretta
TCellManualCorrection	Identification of T cells	Carlo A. Beretta
DrawRectangularROIs	Generation of ROIs	Carlo A. Beretta

Table S3. Composition of collagen matrices

Substance/product	Manufacturer	Volume, µL	Volume, µL
0.2% collagen R solution	Serva, Heidelberg, Germany	90	145
×10 concentrated RPMI-1640 medium supplemented by 2% BSA and 10 mM HEPES	RPMI-1640 (Sigma Aldrich, Steinheim, Germany), BSA and HEPES (Carl Roth)	25.6	20
0.34M sodiumhydroxide	Carl Roth	10.6	12
FITC-conjugated bovine collagen	Sigma-Aldrich	10	10
SiMAG-streptavidin magnetic beads, ∅1 µm, 10 mg/ml	chemicell, Berlin, Germany	5	
Pierce™ Streptavidin magnetic beads, ∅1 µm, 10 mg/ml	ThermoFisher Scientific, Walham, MA, USA		5
Final collagen concentration (mg/ml)		0.95	1.5
